# An additional method for the prevention of hypothermia in severely injured trauma patients

**DOI:** 10.1186/s13054-016-1365-7

**Published:** 2016-07-08

**Authors:** Ellen Omi, Erik Kulstad

**Affiliations:** Department of Surgery and Trauma, Advocate Christ Medical Center, 4440 W. 95th St., Oak Lawn, IL 60453 USA; Department of Emergency Medicine, Advocate Christ Medical Center, 4440 W. 95th St., Oak Lawn, IL 60453 USA

We congratulate Perlman et al. [1] for their excellent manuscript offering a comprehensive review of hypothermia in trauma patients and proposing a goal-directed algorithm for warming the severely injured patient that can be directly incorporated into current Advanced Trauma Life Support guidelines. In this well-crafted algorithm, the authors reveal a pragmatic treatment strategy that can be implemented in most major trauma centers, and that will be evaluated for efficacy in an upcoming prospective trial based in Toronto, ON, Canada.

Although the warming strategies included in this algorithm include a broad range of available techniques and technologies, one approach was omitted (probably due to the fact that it has only recently become available): esophageal heat transfer using a newly available device. In contrast to more traditional lavage, this new device on the market incorporates a closed system of water flow that provides an effective transfer of heat in the efficient heat transfer environment of the esophagus. Recent data have shown success with this device in preventing inadvertent perioperative hypothermia in challenging burn trauma patients [2], as well as in reversing intentionally-induced hypothermia [3]. Ongoing clinical studies are further elucidating the exact quantities of heat transfer capable via this route (ClinicalTrials.gov NCT02743884). Since existing heat exchangers (water blanket chillers) already present in most hospitals are used to power this device, no additional capital expenditures are necessary, making it a cost-effective option. Although initially used primarily for intentional cooling [4], the utility of this approach in trauma patients to provide core warming in a relatively non-invasive manner may warrant its inclusion in subsequent iterations of this well-thought-out algorithm.
